# Capsule Neural Networks with Bayesian Optimization for Pediatric Pneumonia Detection from Chest X-Ray Images

**DOI:** 10.3390/jcm14207212

**Published:** 2025-10-13

**Authors:** Szymon Salamon, Wojciech Książek

**Affiliations:** Department of Computer Science, Faculty of Computer Science and Mathematics, Cracow University of Technology, 31-155 Cracow, Poland

**Keywords:** capsule neural networks, pediatric pneumonia diagnosis, chest X-ray imaging, Bayesian optimization, deep learning in medical imaging

## Abstract

**Background:** Pneumonia in children poses a serious threat to life and health, making early detection critically important. In this regard, artificial intelligence methods can provide valuable support. **Methods:** Capsule networks and Bayesian optimization are modern techniques that were employed to build effective models for predicting pneumonia from chest X-ray images. The medical images underwent essential preprocessing, were divided into training, validation, and testing sets, and were subsequently used to develop the models. **Results:** The designed capsule neural network model with Bayesian optimization achieved the following final results: an accuracy of 95.1%, sensitivity of 98.9%, specificity of 85.4%, precision (PPV) of 94.8%, negative predictive value (NPV) of 96.2%, F1-score of 96.8%, and a Matthews correlation coefficient (MCC) of 0.877. In addition, the model was complemented with an explainability analysis using Grad-CAM, which demonstrated that its predictions rely predominantly on clinically relevant pulmonary regions. **Conclusions:** The proposed model demonstrates high accuracy and shows promise for potential use in clinical practice. It may also be applied to other tasks in medical image analysis.

## 1. Introduction

Although pneumonia is a well-known disease, it remains a major threat to public health, causing approximately 450 million cases and up to 4 million deaths each year worldwide [1]. Children are particularly vulnerable, with an estimated 740,000 pediatric deaths in 2019 alone [2]. Despite the availability of radiological techniques, accurate detection from chest X-rays remains challenging and largely depends on the radiologists’ expertise. These challenges underline the urgent need for computer-aided diagnostic tools to support early and reliable identification of pneumonia in children. The causative agents of pneumonia can be bacterial, viral, and fungal [3]. Furthermore, pneumonia accounts for a significant proportion of all outpatient visits in children, hospital admissions, and antibiotic prescriptions in healthcare facilities in low- and middle-income countries (bacterial pneumonia is the leading cause of severe episodes of pneumonia and deaths from pneumonia), and thus, places a heavy burden on these facilities and the families involved [4]. Recent studies have demonstrated the substantial economic burden of this disease across diverse settings. In Germany, the mean inpatient cost per episode of pneumococcal pneumonia in children reached EUR 2606 with an average hospital stay of 11 days [5]. A global systematic review involving more than 95,000 children under five estimated that severe pneumonia treatment in LMICs costs between USD 242.7 and USD 559.4 per hospitalization, equaling up to 116% of a household’s monthly income [6]. Similarly, a recent multiregional study in China found that the average hospitalization cost for children with pneumonia was 6096 CNY, with total costs reaching 7783 CNY per episode [7]. Together, these findings underscore the considerable clinical and economic burden of childhood pneumonia as well as the pressing need for more effective diagnostic and management strategies. Several radiological techniques are used to diagnose this disease, including chest X-rays, computed tomography, and magnetic resonance imaging. As an easily accessible, painless, and noninvasive examination method, chest X-rays are among the most commonly used radiological examinations for screening and diagnosing various lung diseases. However, the detection of pneumonia based on chest X-rays still largely depends on the diagnostic level of the radiologist, and the reliability of the diagnostic results continues to face enormous challenges [8]. It is, therefore, worth exploiting the growing potential of machine learning methods, in particular neural networks, for the classification of medical data. These algorithms can be applied to various types of data, including medical images such as chest X-rays [9]. These techniques are constantly evolving and allow for increasingly better results in medical diagnostics in the broad sense [10,11,12].

The primary motivation for this research was the need to develop new and effective machine learning models capable of assisting radiologists and physicians in diagnosing pediatric pneumonia from chest X-ray images, thereby reducing the risk of misdiagnosis. Pediatric chest radiographs present unique challenges due to anatomical and developmental differences compared with adults, such as the variable appearance of the thymus in infancy and the frequent presence of congenital anomalies that may mimic or obscure pneumonia on imaging [13]. Moreover, children’s increased radiosensitivity and longer life expectancy necessitate stricter adherence to low-dose protocols and careful collimation to minimize radiation exposure while maintaining diagnostic quality [14]. Accurate interpretation is further complicated by the dynamic nature of pediatric anatomy, where normal variants can resemble pathology, requiring dedicated pediatric imaging protocols and specialized expertise [15]. These specificities underline the importance of developing machine learning models trained explicitly on pediatric pneumonia datasets, rather than extrapolating from adult imaging, to achieve reliable diagnostic support tailored to this vulnerable population.

The key innovation of our study lies in the application of capsule neural networks—originally proposed by Hinton as an alternative to convolutional networks—combined with advanced Bayesian optimization techniques to construct an efficient classification model. Capsule networks have been proposed as an alternative to CNNs because they better preserve spatial hierarchies and part–whole relationships in medical images. At the same time, Bayesian optimization has been widely recognized as an effective strategy for tuning complex deep learning models [16,17]. Unlike prior CNN-based pediatric pneumonia approaches, our method combines these two advantages, ensuring that relevant spatial patterns are retained while hyperparameters are optimized efficiently. This integration provides a more distinctive and reliable framework for analyzing pediatric chest radiographs. We compared our results with existing studies in the literature, and the proposed model demonstrates very high accuracy and sensitivity, substantially lowering the likelihood of missed pneumonia cases.

This article is organized as follows: Section 1 introduces the background and motivation of the study. Section 2 provides a review of the literature. Section 3 describes the dataset, image preprocessing algorithms, capsule neural networks, and Bayesian optimization using the Optuna framework. Section 4 presents the experimental results. Section 5 provides a discussion, highlights the main contributions, compares the findings with related work, and outlines potential directions for future research. Finally, Section 6 concludes the study.

## 2. Literature Review

Artificial intelligence methods, particularly machine learning and neural networks, have achieved remarkable success in medical diagnostics over the past few years. Across nearly all areas of medicine, algorithms have been developed that demonstrate high accuracy in disease prediction, in some cases even surpassing the performance of human diagnosticians and physicians. Machine learning techniques have been applied to the analysis of ECG signals [18], EEG signals [19], the prediction of cancer patient survival [20], and the interpretation of medical images [21]. More recently, models dedicated to predicting pneumonia in children from chest X-ray images have also been proposed. These approaches have employed classical machine learning algorithms, various neural network architectures, advanced preprocessing techniques, feature extraction methods, and optimization strategies. Recent approaches to pediatric pneumonia classification highlight specific methodological strengths rather than relying on generic categories. Transfer learning strategies have proven particularly effective in small and imbalanced pediatric datasets by leveraging pretrained knowledge from larger adult image corpora. Ensemble models, on the other hand, enhance robustness and reduce the variance of predictions by combining complementary classifiers. While earlier studies often employed handcrafted features, deep features automatically learned by neural networks now demonstrate superior performance in capturing subtle radiographic patterns. Taken together, these insights suggest that model selection should be guided by data availability and the trade-off between interpretability and predictive power. Below, we present results from selected scientific studies in this area to illustrate these comparative strengths. In [22], a convolutional neural network based on the VGG16 architecture was employed for pneumonia prediction. The model achieved a classification accuracy of 90.54%, a recall of 98.7%, and an F1-score of 92.9%. In [23], the authors evaluated several pretrained convolutional network architectures, including VGG16, VGG19, ResNet50, and Inception-v3. Among them, VGG16 obtained a classification accuracy of 87.18%, a recall of 96%, and an F1-score of 90%. The VGG19 model reached an accuracy of 88.46%, a recall of 95%, and an F1-score of 91%. By contrast, ResNet50 achieved an accuracy of 77.56%, a recall of 97%, and an F1-score of 84%, while Inception-v3 produced lower performance with an accuracy of 70.99%, a recall of 84%, and an F1-score of 78%. Although deeper architectures such as ResNet50 and Inception-v3 are powerful in large-scale image recognition, their complexity makes them prone to overfitting when trained on relatively small pediatric pneumonia datasets. By contrast, the shallower VGG networks appear to generalize more effectively in this setting, which may explain their comparatively higher accuracy. The observation of high recall but lower precision suggests that some models are more sensitive to pneumonia cases but at the cost of increased false positives. Clinically, this means that while fewer sick children are missed, more healthy patients could undergo unnecessary follow-up tests or interventions. Such trade-offs highlight the importance of balancing sensitivity and specificity when evaluating deep learning models for pediatric diagnosis. Another work [8] proposed a model consisting of 49 convolutional layers and 2 dense layers, which achieved strong performance with an accuracy of 90.5%, a recall of 96.7%, and an F1-score of 92.7%. Ref. [24] introduced an ensemble model that combined two convolutional networks, RetinaNet and Mask R-CNN. This model obtained a recall of 79.3%, an F1-score of 77.5%, and a precision of 75.8%, demonstrating the potential of ensemble approaches for pneumonia prediction. Another highly effective ensemble model was presented in [25], where DenseNet169, MobileNetV2, and Vision Transformer were combined with fine-tuning techniques. The final ensemble, referred to as EL, achieved a classification accuracy of 93.91%, an F1-score of 93.43%, and a recall of 92.99%. Similarly, ref. [9] proposed an ensemble consisting of three convolutional networks with relatively simple architectures, emphasizing efficiency. The networks differed in filter size and threshold values, and their aggregated predictions achieved an accuracy of 84.12%, a recall of 99.23%, and an F1-score of 88.56%. In [26], attention was once again focused on convolutional architectures, where a pretrained and modified Attention-Based DenseNet achieved excellent results with an accuracy of 92.8%, a recall of 96.52%, and an F1-score of 94.3%. A hybrid approach was presented in [27], combining a CNN with the XGBoost algorithm and incorporating data augmentation techniques. The resulting CNN-XGBoost model reached an accuracy of 87%, an F1-score of 87%, and a recall of 85%. Machine learning models have also been successfully applied to the predict various other diseases from chest radiographs, including COVID-19 [28], tuberculosis [29], and rib fractures [30]. This body of work highlights the substantial potential of machine learning in medical diagnostics based on chest X-ray imaging, while also emphasizing the need for further research to develop reliable models that can be effectively integrated into clinical practice. While these results are promising, several barriers remain before such systems can be routinely adopted in clinical settings. The generalizability of models across diverse patient populations and imaging devices remains limited, raising concerns about robustness. In addition, the black-box nature of many deep learning methods challenges interpretability, which is essential for clinical acceptance. Computational cost and infrastructure requirements may also restrict implementation in resource-constrained healthcare environments. Beyond these technical limitations, ethical and legal aspects such as accountability for diagnostic errors, compliance with data protection regulations (e.g., GDPR), and ensuring cybersecurity of medical imaging systems are critical factors that must be addressed before widespread clinical deployment. In our research, we intend to employ capsule neural networks together with Bayesian optimization in order to obtain superior results compared to those reported in the literature, or comparable results using less complex architectures, which will in turn accelerate the training and prediction phases.

## 3. Materials and Methods

### 3.1. Dataset

For the purposes of this study, we used the well-known, publicly available dataset Chest X-ray Images (Pneumonia), hosted on the Kaggle platform [31]. The dataset contains 5863 chest radiographs divided into two categories: normal (healthy patients) and pneumonia (patients diagnosed with pneumonia). The images represent pediatric patients between one and five years of age and were collected at the Guangzhou Women and Children’s Medical Center in Guangzhou. All images are stored in JPEG format, with dimensions of approximately 1024 × 1024 pixels. In this study, the images were subjected to the required preprocessing prior to their use in model training.

**Figure 1:**
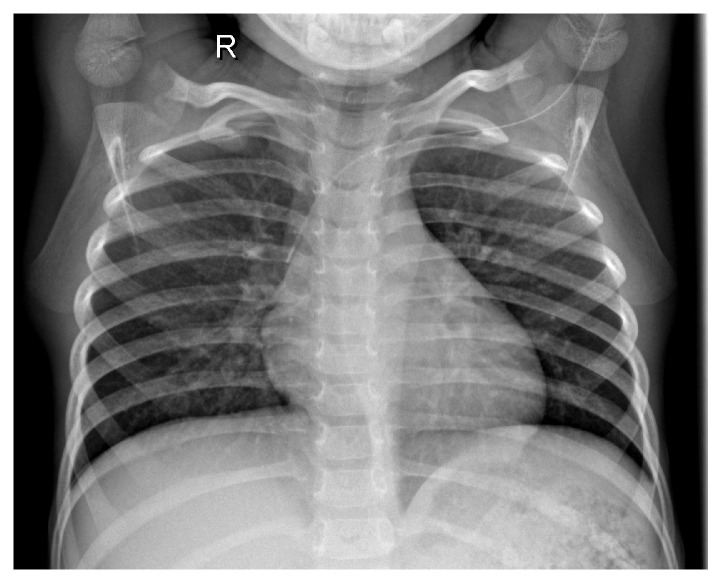
Healthy patient X-ray.

Figure 1 shows the X-ray of a healthy patient.

**Figure 2:**
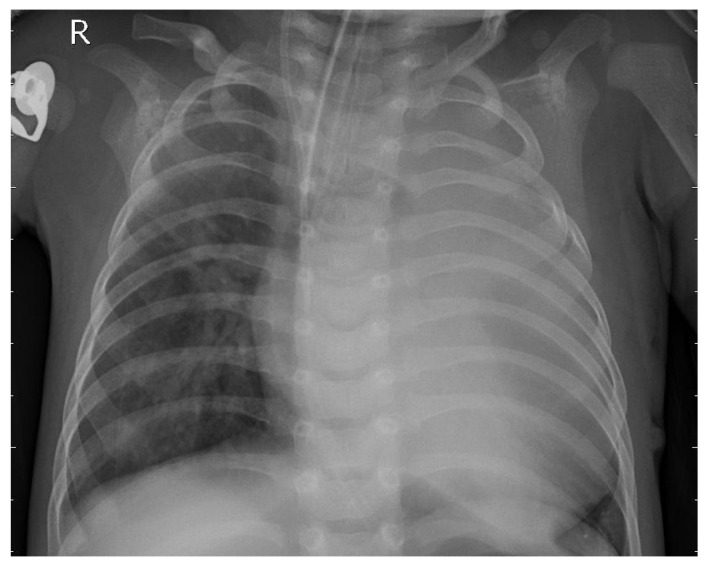
Bacterial pneumonia patient X-ray.

Figure 2 depicts the chest X-ray of a patient diagnosed with pneumonia.

### 3.2. Preprocessing

The images were rescaled to dimensions of 224 × 224 pixels using bilinear interpolation. Pixel intensity values were then normalized to the range 0–1. In the subsequent step, noise reduction was performed using a Gaussian filter, followed by the application of the CLAHE algorithm [32] and gamma correction.

**Figure 3:**
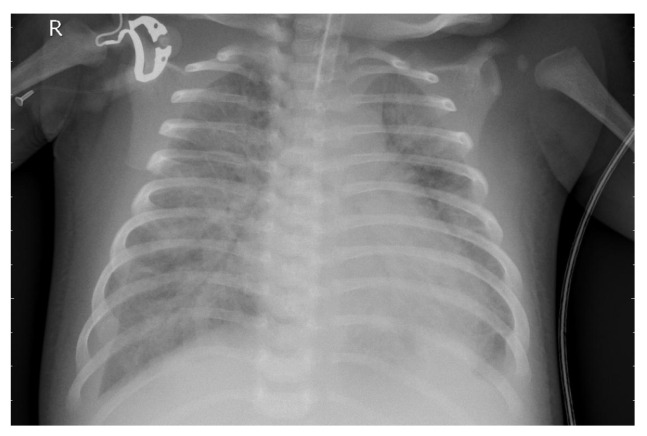
Original X-ray image.

Figure 3 presents the X-ray image before preprocessing was applied.

**Figure 4:**
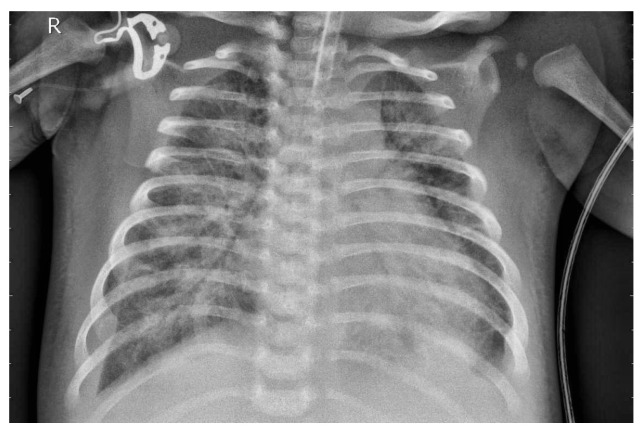
X-ray image after preprocessing.

Figure 4 presents the image following the necessary preprocessing steps.

Preprocessing of medical images, such as X-ray scans, plays a crucial role in providing high-quality input data for machine learning models. These steps allow models to focus on clinically relevant features rather than artifacts. As a result, the overall robustness and accuracy of the predictive algorithms are improved. Therefore, effective preprocessing is a key prerequisite for the reliable application of deep learning in medical image analysis.

### 3.3. Capsule Neural Networks

Capsule neural networks represent an extension of convolutional neural networks. They were first introduced in 2017 [33]. Since the first publication CapsNets demonstrated superiority over classical convolutional networks in image recognition tasks. Just one year later, Afshar adapted the prototype CapsNet for medical image analysis, showing the fact that the model was able to preserve the precise location of shaded regions and achieved sensitivity several percentages points higher compared to the convolutional model. Capsule networks extend the idea of convolutional neural networks by replacing the traditional scalar neuron with a capsule, represented as a vector or matrix. A capsule encodes not only the presence of a feature but also properties such as orientation, position, and scale, enabling the hierarchical representation of images structures. In the original architecture, the PrimaryCaps layer convolutionally transform feature maps into vectors, where the length of the vectors corresponds to the probability of existence of a feature and the orientation of the vector encodes its attributes. The higher-level capsule (DigitCaps) then aggregates these features through a dynamic routing mechanism, which iteratively adjusts the routing weights based on the level of agreement between the predictions of the lower-level capsules and the activations of the higher-layer capsules [34]. The dynamic routing process ensures that capsules focus on connections best representing the hierarchical structure of the input, with vector aggregation regulated by the squash function that normalizes their length. Compared to conventional CNNs, CapsNet preserves spatial relationships between anatomical structures by avoiding pooling operations that reduce feature resolution. This property is particularly important in pediatric chest radiographs, where anatomical proportions differ from those of adults and subtle variations may indicate pneumonia. By modeling part–whole hierarchies more effectively, CapsNet provides greater robustness to positional and geometric variations commonly found in clinical imaging. These characteristics make it especially suitable for supporting reliable detection of pediatric pneumonia in chest X-ray images.

Capsule neural networks have already been applied to medical imaging tasks, including lung tomography segmentation and the delineation of muscle and fat tissue in thigh MRI scans [35]. They have also demonstrated utility in monkeypox classification [36], brain image segmentation [37], and COVID-19 detection [38].

### 3.4. Optuna

For many years, the dominant algorithms used for hyperparameter selection in machine learning models have been random search and grid search. Recent studies, however, suggest that Bayesian optimization can achieve superior results with fewer evaluations. Unlike exhaustive or purely random approaches, Bayesian optimization constructs a probabilistic model of the objective function and iteratively selects hyperparameter configurations that are most likely to improve performance. This makes it particularly effective for computationally expensive models, such as deep neural networks, which require considerable time and resources for each evaluation. These algorithms have been implemented and made available in libraries such as Optuna [39], which has been successfully applied in numerous scientific studies [20,40,41]. In our research, Optuna was employed to optimize the hyperparameters of the neural network.

Table 1 presents the hyperparameters optimized using the Optuna framework. The number of trials was set to 100.

### 3.5. Proposed Model

Before implementing the model, analysis and design procedures were performed to guide the selection of appropriate layers and hyperparameters. Chest X-ray images exhibit a significant brightness variation and slight differences between pathologic and nonpathologic tissues. While CNNs are efficient in learning local features, they fail to preserve orientation and spatial relationships. For this reason, a CapsNet-based architecture was chosen, where capsules have the capability to encode the existence and geometric properties of features. The model begins with a convolutional block with ReLU activation and pooling that is responsible for capturing low-level feature maps such as textures and edges. The outputs of this are fed to the PrimaryCaps layer, which encodes local information into eight-dimensional vectors. With stride 2, the spatial resolution becomes downsampled to 56 × 56, and 32 capsules with channels offer a dense feature representation. The DigitCaps layer dynamically routes, which produces two 16-dimensional output capsules for the Normal and Pneumonia classes. It makes three iterations to strengthen stable connections between lower- and higher-level capsules. Consistent with [33], we used three routing iterations as an empirically validated compromise between convergence stability, accuracy, and computational efficiency. For training, the margin loss function was used using the 0.9 and 0.1 threshold and the balance factor λ=0.5 so that the lengths of the capsules represent the probabilities of classes. This configuration follows the original formulation in [33], where the parameters were empirically validated as an effective balance between promoting the activation of the correct class capsule and suppressing incorrect ones. Optimization was performed using Adam optimizer at the learning rate 1 × 10^−4^ to have stable convergence of routing-based gradients. Implementation within Keras combined standard modular layers with new capsule elements. A nonlinear squash function was defined to normalize capsule vectors without orientational loss as a feature information carrier. The PrimaryCaps layer was employed as a convolution and generated K × dK channels, reshaped into capsules, while DigitCaps employed transformation matrices and iterative routing to output class-level capsules. The final capsule lengths formed the direct classification probabilities, and they offered superior spatial feature representation compared to the traditional CNNs.

**Figure 5:**
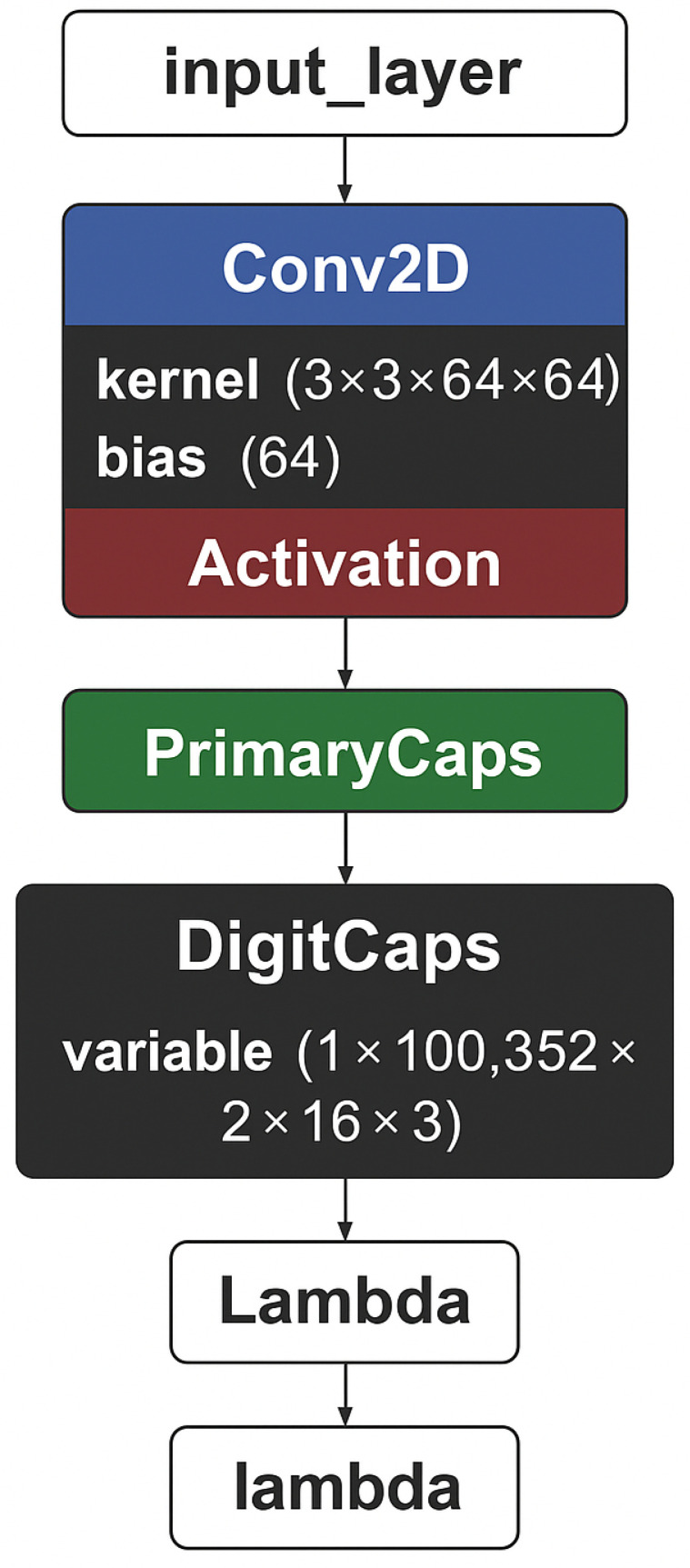
Proposed architecture schema.

Figure 5 presents the architecture of our capsule neural network model.

Table 2, in contrast, presents further details, such as the number of parameters for each layer.

The proposed architecture is comparatively less complex, comprising substantially fewer layers than many pretrained models, while still maintaining high classification accuracy.

### 3.6. Software and Hardware Configuration

In this study, the algorithms were implemented in the Python (version 3.8.10) programming language. Several widely used professional libraries were employed, including NumPy (version 1.24.3) [42], OpenCV (version 4.12.0) [43], TensorFlow (version 2.13.0) [44], scikit-learn (version 1.3.2) [45], and Matplotlib (version 3.7.2) [46]. The computational environment was managed using Conda and Anaconda Navigator [47], while the source code was developed in Visual Studio Code (version 1.105.0). Regarding the hardware setup, the neural network was trained on an NVIDIA RTX 5070 Ti graphics card with 16 GB of VRAM. The system was further equipped with an AMD Ryzen 9 7900X and 32 GB of RAM.

### 3.7. Metrics

In the conducted experiments, standard metrics commonly used for evaluating machine learning models were employed. Their formulas are presented below.

- Accuracy measures the proportion of correctly classified samples among all samples:
  (1) $$\mathrm{Accuracy}=\frac{TP+TN}{TP+TN+FP+FN}$$
- Recall (Sensitivity, True Positive Rate) quantifies the proportion of correctly identified positive cases:
  (2) $$\mathrm{Recall}=\frac{TP}{TP+FN}$$
- Specificity (True Negative Rate) quantifies the proportion of correctly identified negative cases:
  (3) $$\mathrm{Specificity}=\frac{TN}{TN+FP}$$
- Precision (Positive Predictive Value, PPV) measures the proportion of correctly predicted positive cases among all predicted positives:
  (4) $$\mathrm{PPV}=\frac{TP}{TP+FP}$$
- Negative Predictive Value (NPV) measures the proportion of correctly predicted negative cases among all predicted negatives:
  (5) $$\mathrm{NPV}=\frac{TN}{TN+FN}$$
- False Positive Rate (FPR) measures the proportion of negative cases incorrectly classified as positive:
  (6) $$\mathrm{FPR}=\frac{FP}{FP+TN}$$
- F1-score is the harmonic mean of precision and recall:
  (7) $$F1=\frac{2\cdot Precision\cdot Recall}{Precision+Recall}$$
- Area Under the Curve (AUC) refers to the area under the Receiver Operating Characteristic (ROC) curve, which illustrates the trade-off between the True Positive Rate and the False Positive Rate. AUC values range from 0 to 1, with higher values indicating better discrimination performance.
- Matthews Correlation Coefficient (MCC) provides a balanced measure that can be used even if the classes are of very different sizes:
  (8) $$MCC=\frac{TP\cdot TN-FP\cdot FN}{\sqrt{\left(TP+FP)(TP+FN)(TN+FP)(TN+FN\right)}}$$

### 3.8. Explainability Analysis

Artificial intelligence algorithms are increasingly successful in medical applications. However, their introduction into clinical practice raises many challenges, particularly those related to understanding the models and building trust in them. This is especially relevant for models such as support vector machines or deep neural networks, whose internal mechanisms remain largely opaque to end-users. This lack of transparency amplifies concerns about potential harm, bias, fairness, autonomy, legal accountability, and physician–patient communication [48]. Therefore, the use of model explainability algorithms in artificial intelligence research is of utmost importance. Among the most popular approaches are methods such as SHAP values [49] and LIME [50]. However, their applications are mostly limited to tabular data [51]. In the case of imaging data, the most commonly used method is Grad-CAM [52], which has become the de facto standard for medical images. Grad-CAM works by backpropagating the gradients of a target class to the last convolutional layers of the network, thereby producing a coarse localization map that highlights the most relevant regions of the input image. This allows researchers and clinicians to verify whether the model is focusing on meaningful anatomical structures when making predictions. In our study, we applied this method to validate the behavior of our final model on the test data.

## 4. Results

This section may be divided by subheadings. It should provide a concise and precise description of the experimental results, their interpretation, as well as the experimental conclusions that can be drawn. In this section, we present the results of our study. The primary objective was to design a machine learning algorithm based on capsule neural networks in order to achieve high accuracy in predicting pneumonia in children. The experiments were conducted using the publicly available Chest X-ray Images (Pneumonia) dataset. The study was carried out in two stages. In the first stage, we applied the designed capsule neural network model. In the second stage, the research was extended by incorporating Bayesian optimization to fine-tune the hyperparameters of the network. The models were evaluated using standard performance metrics, including Accuracy, F1-score, Recall, Specificity, PPV, NPV, FPR, AUC, and MCC. In addition, we present the learning curves of the neural network, confusion matrices, and ROC curves. Such a comprehensive evaluation provides a realistic assessment of the model’s potential for clinical application.

### 4.1. Capsule Neural Network Model

**Figure 6:**
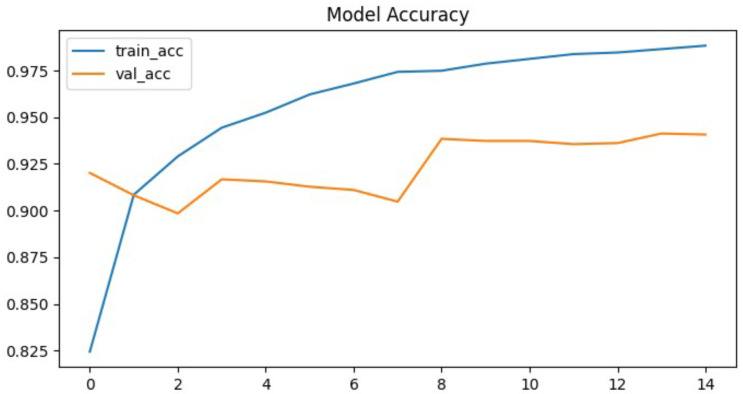
Proposed architecture model accuracy.

**Figure 7:**
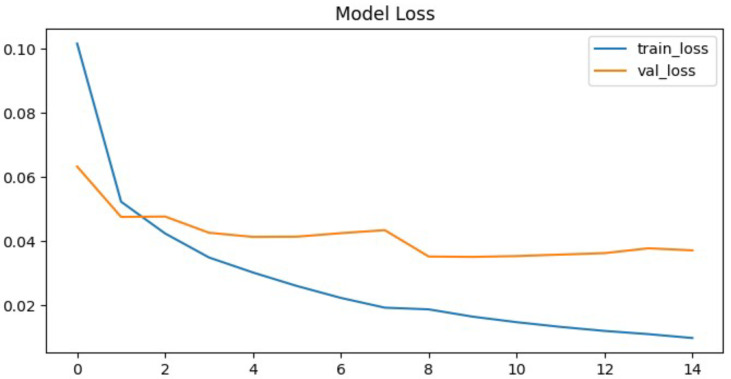
Proposed architecture model margin loss.

To further evaluate the performance of the proposed neural network, the training process was monitored using accuracy and loss metrics. Figure 6 and Figure 7 illustrate the model’s accuracy and margin loss across training and validation sets over 14 epochs. These plots provide insights into the convergence behavior of the network, the stability of learning, and the degree of generalization to unseen data.

The proposed neural network demonstrated rapid convergence during training, which was clearly reflected in both the accuracy and the loss curves. Training precision exhibited a steep increase, rising from approximately 82% in the initial epoch to almost 99% in the ninth epoch. This rapid convergence can be attributed to the relatively smaller parameter space of the CapsNet architecture compared to deeper CNNs, which reduces the risk of overfitting and allows faster optimization. In addition, hyperparameter tuning with Optuna likely facilitated more effective learning rate adaptation, further supporting efficient training dynamics. After this point, the curve reached a plateau, maintaining high performance without further substantial improvements. This indicates that the network quickly adapted to the training data and effectively minimize classification errors.

In contrast, the validation set accuracy followed a less stable trajectory. Although it reached its maximum value of 94% at epoch nine, subsequent epochs were characterized by oscillations rather than steady improvement. By epoch fourteen, when the training process was terminated due to the early stopping criterion, the validation accuracy stabilized at 94.1%, essentially equivalent to the best value observed earlier. The discrepancy between the steadily increasing training accuracy and the fluctuating validation accuracy is a clear signal of overfitting, where the model continued to specialize on the training data but was unable to achieve corresponding improvements on unseen validation samples. This overfitting can likely be attributed to the relatively limited size of the pediatric chest X-ray dataset, which constrained the model’s ability to generalize. Furthermore, the expressive capacity of CapsNet may have exceeded the available training data, leading to memorization of patterns rather than robust feature learning. The lack of stronger regularization techniques, such as additional dropout layers or more aggressive data augmentation, may also have contributed to the observed fluctuations in validation accuracy. A more systematic investigation of these factors, including dataset expansion and enhanced regularization strategies, represents an important direction for future research.

A similar trend can be observed in the margin loss curves. The training loss decreased sharply, dropping from an initial value of approximately 0.1 to around 0.012 by the later epochs. This reduction highlights the efficiency of the optimization process and confirms that the model successfully minimized the chosen objective function. However, the validation loss did not follow the same pattern. Instead of decreasing consistently, it oscillated around higher values and never dropped below 0.04. After epoch nine, the validation loss remained stagnant, again pointing to limitations in the network’s ability to generalize beyond the training set.

Despite these challenges, the model’s final evaluation on the independent test set yielded an accuracy of 93.5%, which is close to the peak validation accuracy. This result demonstrates that, although overfitting occurred, it did not cause significant degradation when applied to previously unseen data. The test set contained 880 chest radiographs, which represents a relatively large and representative subset of the dataset. Therefore, the reported accuracy values can be considered reliable and not strongly influenced by random variance.

### 4.2. Capsule Neural Network Tuned with Bayesian Optimization

To further improve the generalization of the capsule network and mitigate overfitting, the model was fine-tuned using Bayesian optimization with the Optuna framework. Capsule networks combine a large number of architectural and training hyperparameters, including capsule dimensions, routing iterations, learning rate, batch size, and dropout rate, resulting in a vast multidimensional search space that is inefficient to explore manually. Optuna addresses this challenge by leveraging Bayesian optimization with accumulated knowledge from previous trials, rapidly narrowing the search toward the most promising hyperparameter regions. Additionally, the built-in pruning mechanism terminates poorly performing trials early, significantly reducing computational cost.

For this study, the search space included the learning rate, the number of filters, the dropout rate, the batch size, and the dimensions of the capsule. To accelerate the optimization process, the input image resolution was reduced from 224 × 224 to 112 × 112 pixels, and training was limited to 40% of the full dataset. Each trial was trained for a maximum of 8 epochs, and the optimization procedure was constrained to 100 trials or 8 h of runtime, whichever occurred first. The pruning mechanism cut short 12% of the weakest trials, saving an estimated two hours of training time.

The best trial achieved 93% accuracy within the constraints of the optimization setting. Although this result was slightly below the original baseline, it should be emphasized that the reduced training depth and the fraction of the dataset limited the achievable performance. The identified hyperparameters were subsequently used for full training of the model. In this final run, the early stopping criterion was reached at epoch 25, primarily due to the reduced learning rate, which allowed for finer weight adjustments and more stable convergence.

The training curves show that the optimized model exhibited smoother and more stable learning dynamics compared to the baseline. Accuracy increased gradually rather than converging too rapidly, and validation accuracy closely followed training accuracy throughout the process. The best performance was achieved at epoch 21, with a validation accuracy of 95.2%, while the test set reached an accuracy of 95.1%. Training accuracy peaked at 94.85% by epoch 25, demonstrating a well-balanced fit between training and validation data. Importantly, the use of dropout at 30% effectively mitigated overfitting, as evidenced by the parallel decrease of both training and validation loss curves.

A confusion matrix provides further information on classification performance. Out of all test samples, the model made 35 false positive predictions, where healthy chest X-rays were misclassified as pneumonia, and 8 false negatives, where pneumonia cases were misclassified as normal. From a clinical perspective, this outcome is favorable: false positives lead to additional diagnostic checks, while minimizing false negatives reduces the risk of overlooking actual pathological cases. Based on the confusion matrix, the sensitivity (recall) for pneumonia was calculated at approximately 99%, confirming that nearly all diseased cases were correctly identified, while the sensitivity for normal cases was 85%.

In conclusion, Bayesian optimization with Optuna successfully identified hyperparameters that improved the stability and generalization of the capsule network. Compared to the baseline model, the optimized network achieved a +2.5% higher validation accuracy and a +1.5% higher test accuracy, while maintaining balanced error distribution between classes. The combination of reduced learning rate and dropout regularization was particularly effective in preventing overfitting, demonstrating the value of automated hyperparameter search for computationally demanding models such as capsule networks.

**Figure 8:**
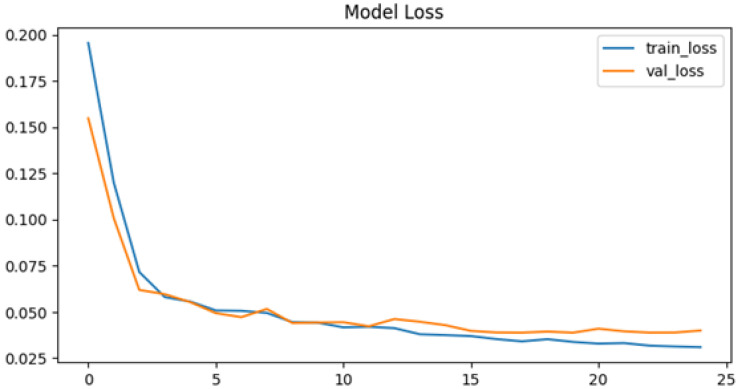
Model loss after tuning.

As shown in Figure 8, both training and validation loss decrease smoothly throughout training. The fact that the validation loss closely follows the training loss indicates good generalization after hyperparameter tuning. The stabilization at low values demonstrates effective convergence.

**Figure 9:**
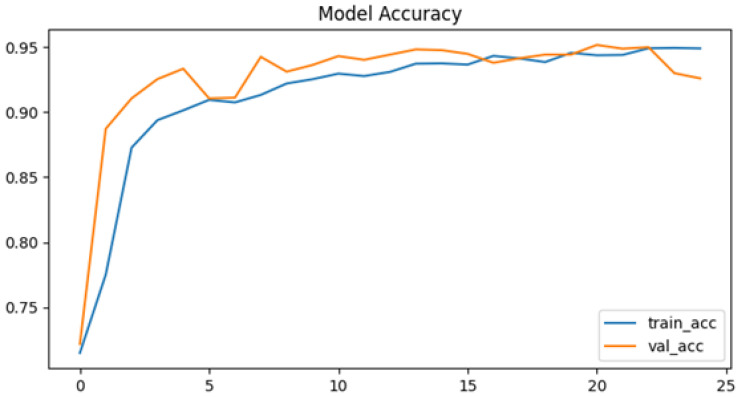
Model accuracy after tuning.

In the accuracy plot (Figure 9), the training and validation curves progress in parallel, indicating stable learning dynamics. Validation accuracy peaks at 95.2%, closely matching training accuracy, which shows that the tuned model avoided significant overfitting. The plateau after epoch 20 highlights convergence.

**Figure 10:**
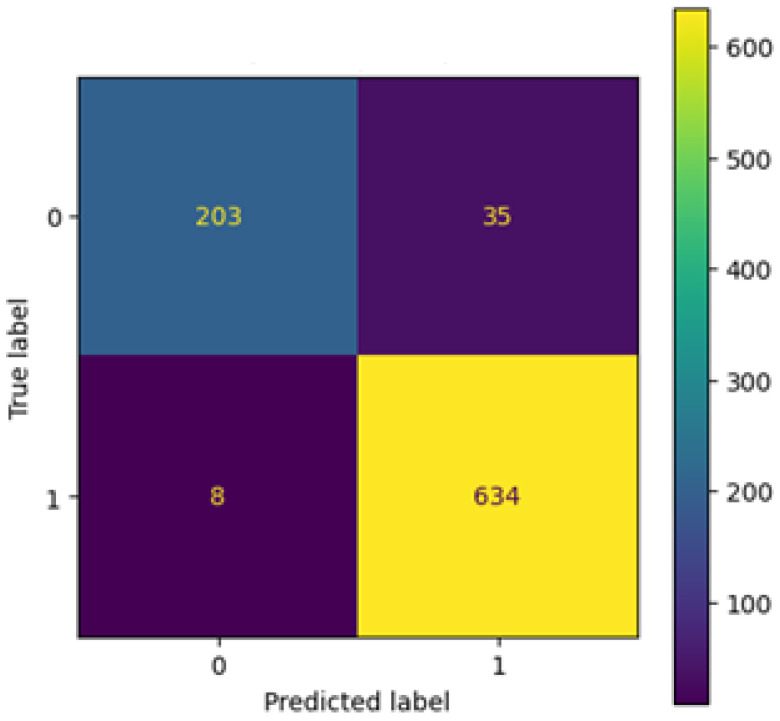
Confusion matrix after tuning.

The confusion matrix (Figure 10) indicates that the model accurately classifies the vast majority of samples. Only 35 false positives and 8 false negatives are visible, resulting in a high recall rate for pneumonia cases (99%). This demonstrates strong clinical relevance, as nearly all diseased cases were detected.

**Figure 11:**
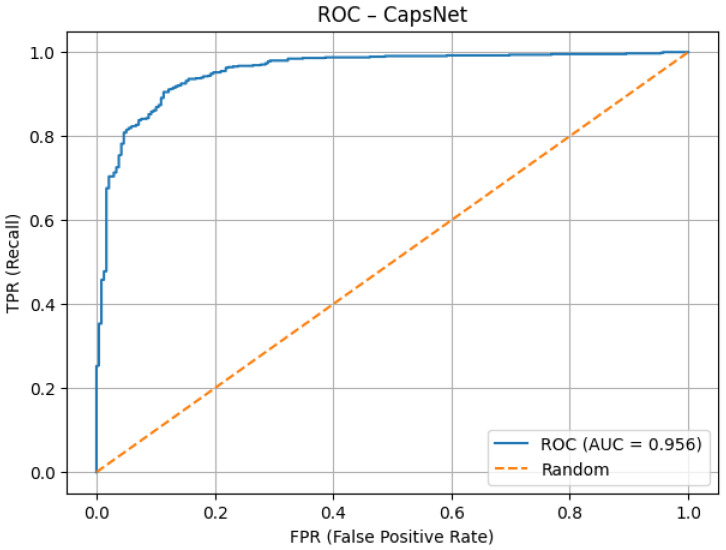
ROC AUC after tuning.

As illustrated in the ROC curve (Figure 11), the model achieves excellent discrimination ability, with an AUC score of 0.956. The steep rise toward the upper-left corner indicates that the network maintains high true positive rates while keeping false positive rates low. This confirms robustness in distinguishing between the two classes.

**Figure 12:**
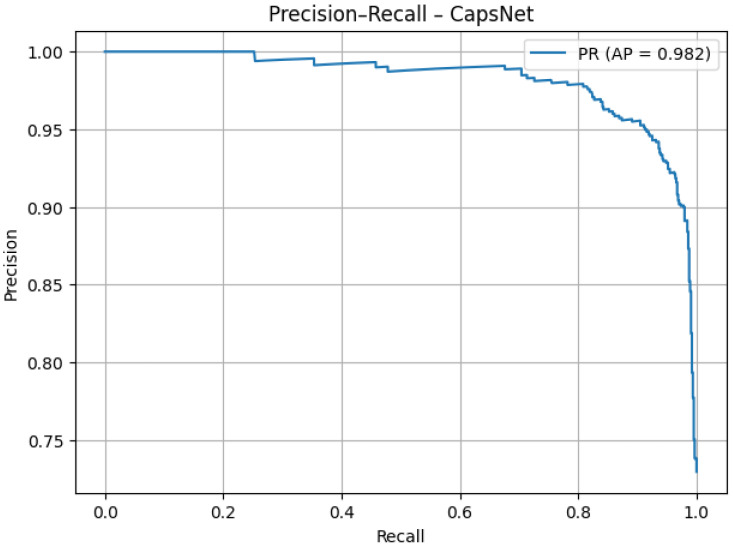
PR AUC after tuning.

The precision–recall curve (Figure 12) demonstrates that the model maintains high precision (>95%) across most recall values. The average precision of 0.982 confirms strong performance in detecting pneumonia cases with minimal false alarms. The slight decline at very high recall values is expected and does not undermine overall effectiveness.

As presented in Table 3, the tuned capsule network achieved an overall accuracy of 95.1%, with a very high sensitivity of 98.7% and a specificity of 85.3%. Precision (94.7%) and negative predictive value (96.2%) further confirm the balanced performance across both classes. The high F1 score (0.967), MCC (0.874), and strong AUC values for both ROC (0.956) and PR (0.982) demonstrate that the optimized model not only generalized well but also maintained clinical reliability by minimizing false negatives while keeping false positives at an acceptable level.

To gain further insight into the decision-making process of our capsule network, we present Grad-CAM visualizations of a representative chest X-ray correctly classified as positive. Grad-CAM highlights the image regions that most strongly influence the model’s output, enabling us to evaluate whether the network focuses on anatomically meaningful structures.

**Figure 13:**
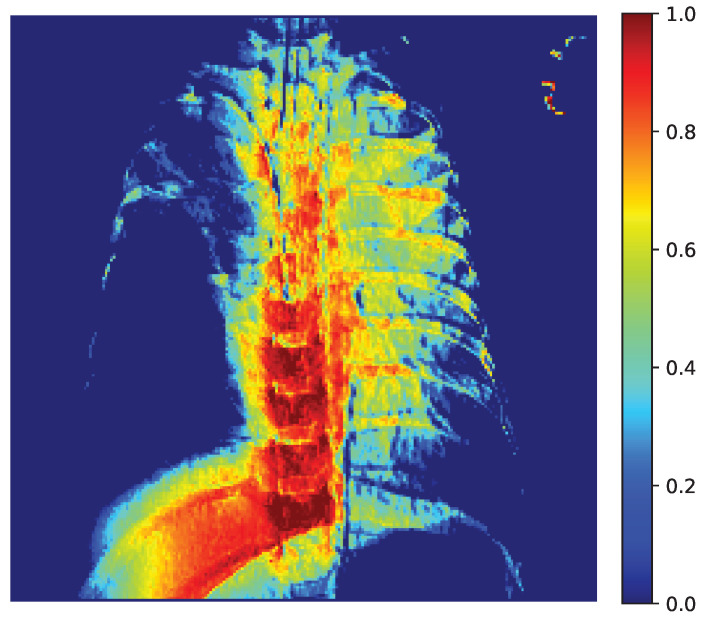
Grad-CAM Activation Map (0–1).

**Figure 14:**
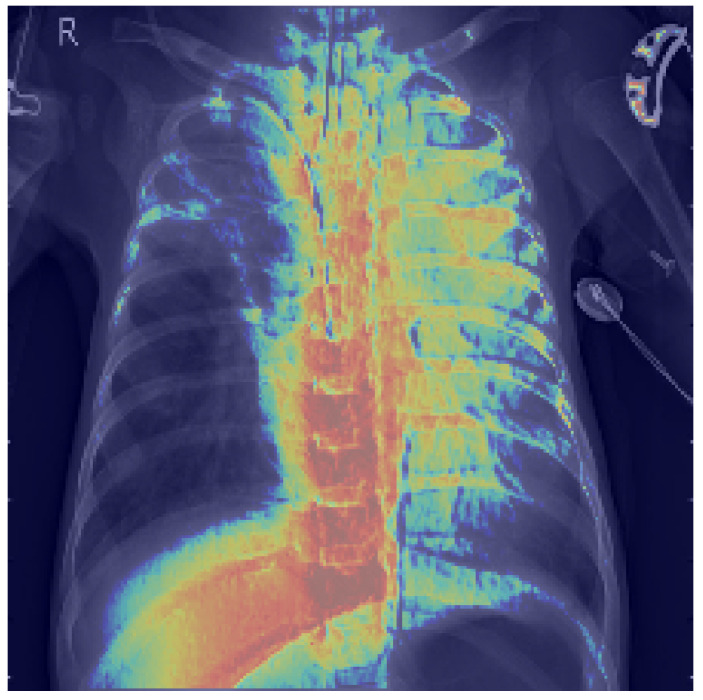
Grad-CAM heatmap overlaid on chest X-ray.

Figure 13 displays the raw activation map (range 0–1), where warmer colors indicate a stronger contribution to the decision. Figure 14 shows the same heatmap superimposed on the original radiograph, aiding anatomical interpretation. The strongest activations are located within the pulmonary fields, particularly around the hila and medial inferior lung zones, with weaker scattered responses along the spine and ribs. This distribution suggests that CapsNet primarily relies on parenchymal density patterns and textural inhomogeneities consistent with the target pathology, whereas extrapulmonary highlights may reflect exposure or contrast effects, osseous contours, or device-related artifacts (e.g., leads, electrodes).

Our quantitative analysis, which computes the share of pixels with Grad-CAM intensity above a threshold within lung masks versus nonlung areas confirms that a substantial proportion of the heatmap energy lies within the lungs. These findings indicate that the model’s decisions are grounded in clinically relevant regions, thereby supporting the reliability of the reported performance.

## 5. Discussion

Despite the continuous advancement of modern medicine, pediatric pneumonia remains a serious threat to child health and life and represents a considerable burden for healthcare systems worldwide. In this study, we propose supporting the diagnosis of this disease through advanced artificial intelligence methods—specifically, capsule networks combined with Bayesian optimization. Our experiments were conducted on the well-known Chest X-ray Images (Pneumonia) dataset, which contains nearly 6000 radiographs of pediatric patients. The primary goal was to develop effective classification models optimized with the Optuna framework while also incorporating model explainability analysis. Previous approaches to pneumonia prediction from chest X-rays have relied predominantly on pretrained convolutional neural networks, whereas our work introduces a novel methodology by integrating capsule networks with Bayesian optimization. The novelty of this approach is reflected in the results obtained, which were evaluated using metrics such as accuracy, F1-score, and recall, and further analyzed through confusion matrices and ROC curves. Beyond performance metrics, our study highlights the potential of tailoring deep learning architectures to the unique anatomical characteristics of pediatric patients, which differ significantly from those from adults. Capsule networks, by modeling part–whole hierarchies, may provide a more faithful representation of pediatric lung structures. The use of Bayesian optimization further demonstrates that systematic hyperparameter search can yield efficient yet robust models, even when datasets are relatively limited. This methodological synergy illustrates how modern AI techniques can be adapted to address practical diagnostic challenges rather than focusing solely on accuracy benchmarks. Finally, the inclusion of explainability analysis underscores our commitment to developing clinically relevant tools that can foster physician trust and facilitate integration into real-world radiology practice.

The proposed capsule network demonstrated a high level of effectiveness in pneumonia detection from chest X-rays, achieving an overall accuracy of 95.1%. Most notably, the model reached a sensitivity of 98.7%, which indicates that nearly all pneumonia cases were successfully identified, with only 1.25% of actual positive cases misclassified as healthy. From a clinical perspective, this property minimizes the risk of missed diagnoses, which is critical for timely medical intervention and reducing mortality. The specificity of 85.3% shows that some healthy cases were classified as pathological, but this trade-off is acceptable in medical contexts where false negatives are far more detrimental than false positives.

The precision of 94.8% confirms that the majority of predicted pneumonia cases corresponded to real disease, while the negative predictive value (NPV) of 96.2% indicates that patients classified as healthy can be safely discharged without additional diagnostic verification. The F1-score of 96.7% demonstrates that the model maintained a strong balance between sensitivity and specificity, which is particularly important given the class imbalance present in the dataset. Furthermore, the Matthews Correlation Coefficient (MCC) of 0.87 underlines the robustness of the model even under conditions of unequal class distribution. Taken together, these results indicate that the capsule network successfully prioritized sensitivity while maintaining acceptable levels of specificity, making it a valuable clinical support tool.

### 5.1. Comparison with State-of-the-Art Approaches

To contextualize the results, the capsule network was compared with other architectures reported in the literature. A detailed comparison is provided in Table 4.

A hybrid CNN + XGBoost model proposed by Hedhoud (2023) [27] achieved 87% accuracy, 85% sensitivity, and 89% specificity, but missed approximately 15% of pneumonia cases. While this approach offers significantly faster inference (∼7 s on the test set), its lower sensitivity reduces its suitability for clinical scenarios where missing diseased cases is unacceptable.

Other CNN-based models typically achieved 92–93% accuracy, 89–91% sensitivity, and 93–94% specificity, demonstrating strong class separation ability but still failing to reach the sensitivity level of the proposed capsule network. In contrast, the presented model achieved the highest accuracy (95%) and sensitivity (98.7%), albeit at the expense of reduced specificity (by 8–9 percentage points). This trade-off is justifiable in clinical practice, as the minimization of false negatives is often the top priority, while false positives can be clarified through additional diagnostic examinations.

Compared to human experts, as reported in the CheXNet study by [53], radiologists achieved an F1 score of approximately 39% on the imbalanced Chest X-ray14 dataset. While direct comparisons are not strictly valid due to differences in dataset composition and class distribution, our capsule network nevertheless achieved a markedly higher F1 score within the pediatric dataset. This suggests that the model is capable of capturing subtle radiographic patterns that can challenge human interpretation. Moreover, the model’s consistency, independence from fatigue or stress, and ability to preprocess images further enhance its potential as a supportive diagnostic aid.

When compared with other capsule-based approaches, the model also performs strongly. Ref. [54] introduced COVID-CAPS, which achieved 95.7% accuracy, 90% recall, and 92.2% F1-score. In contrast, the proposed model achieved higher recall (98.7%) and F1-score (96.7%), highlighting its superior ability to capture pneumonia-related features. Ref. [55] presented the Deep Context Axial Reverse Capsule Network with Sea-Horse Optimizer, which achieved 96.8% accuracy, 93.4% precision, and 95.1% F1-score. While this model attained slightly higher accuracy, the proposed capsule network demonstrated better balance between sensitivity and specificity, and achieved superior overall precision without relying on complex optimization schemes.

### 5.2. Strengths of the Approach

Several strengths of the proposed solution can be highlighted. The combination of dynamic routing and hyperparameter tuning enabled the model to achieve nearly perfect recall, virtually eliminating the risk of missing pneumonia cases. Capsule networks, unlike standard convolutional filters, preserve spatial and hierarchical relationships, which allows for robust generalization even under suboptimal imaging conditions. This is particularly advantageous for pediatric cases, where anatomical variability is greater.

The use of Docker ensured reproducibility and portability of experiments, while Optuna enabled efficient hyperparameter search through Bayesian optimization and pruning. Together, these tools provide a reliable experimental framework, making the model easy to transfer, retrain, or adapt to new datasets. Compared to CNNs, the capsule network offers superior accuracy and recall, while maintaining clinically acceptable specificity, making it a strong candidate for integration into radiological workflows as a supportive diagnostic system.

### 5.3. Limitations and Challenges

Despite these promising results, several limitations remain. Capsule networks are computationally demanding, as dynamic routing requires iterative processing of vectors, resulting in significantly longer training and inference times compared to CNNs. Even with modern GPUs, training was resource-intensive, which may hinder adoption in environments with limited hardware availability. Memory requirements are also higher due to the generation of high-dimensional tensors. Reducing image resolution can alleviate this issue but risks losing diagnostically relevant details.

Another limitation is the lower specificity of the model, which could pose challenges in clinical settings where unnecessary follow-up examinations may burden healthcare systems with limited resources. Additionally, the model was trained on a single dataset, focusing exclusively on children under five years old. This introduces the risk of domain shift and limits the generalizability of the model to other populations. Capsule networks, like other deep learning models, also function as “black boxes,” which raises concerns about interpretability. Tools such as Grad-CAM or attention maps should be incorporated to visualize the decision-making process and support clinical acceptance. Finally, although Optuna streamlined the hyperparameter search, the complexity of capsule architectures creates a vast parameter space with nonlinear interactions, meaning that optimal solutions may still be local rather than global optima.

### 5.4. Future Research Directions

Building on these findings, several research avenues are recommended:

1. Federated learning—Training capsule networks across multiple institutions in a privacy-preserving manner would enable larger and more diverse datasets, reducing bias and improving generalization without compromising patient confidentiality.
2. Multimodal clinical data integration—Incorporating patient metadata (e.g., age, sex, laboratory test results) alongside imaging data could improve diagnostic accuracy and allow the model to predict disease severity or comorbidities.
3. Clinical validation and standardization—Multicenter prospective trials comparing CapsNet with radiologists are essential to assess its impact on clinical workflows. Regulatory certification will also require comprehensive documentation and monitoring systems capable of detecting model drift over time.

## 6. Conclusions

In this study, we designed a novel machine learning model based on capsule neural networks combined with Bayesian optimization. The proposed model achieved high performance, with an accuracy of 95.1% and an F1-score of 96.8%. These results indicate the potential for future integration of the model into clinical practice. Nevertheless, further research is required to develop more advanced models that can provide reliable support for healthcare systems. Artificial intelligence has significant potential to enhance medical image classification, and the proposed approach should also be considered for diagnosing other diseases.

## Figures and Tables

**Table 1:** Hyperparameter search space explored by Optuna.

Hyperparameter	Search Space
lr	from $1\times 10^{-5}$ to $1\times 10^{-3}$ (log scale)
n_filters	{32,64,128}
drop_rate	from 0.0 to 0.5 (continuous)
cap_dim	{8,16,32}
batch_size	{8,16,32}

**Table 2:** Summary of the proposed CapsNet model architecture.

Layer (Type)	Output Shape	Param #
Input_10 (InputLayer)	(None, 224, 224, 3)	0
Conv2D_18 (Conv2D)	(None, 224, 224, 64)	1792
MaxPooling2D_9 (MaxPooling2D)	(None, 112, 112, 64)	0
PrimaryCaps_9 (PrimaryCaps)	(None, 100, 352, 8)	147,712
DigitCaps_9 (DigitCaps)	(None, 2, 16)	25,690,112
Lambda_9 (Lambda)	(None, 2)	0
Total params:		25,839,616 (98.57 MB)
Trainable params:		25,839,616 (98.57 MB)
Nontrainable params:		0 (0.00 Byte)

**Table 3:** Tuned model results.

Metric	Value
Accuracy	0.951136
Sensitivity (Recall)	0.987539
Specificity	0.852941
Precision (PPV)	0.947683
Negative Predictive Value (NPV)	0.962085
False Positive Rate (FPR)	0.147059
False Negative Rate (FNR)	0.012461
F1 Score	0.967201
Matthews Correlation Coefficient (MCC)	0.874438
ROC AUC	0.955797
PR AUC	0.981815

**Table 4:** Comparison of the obtained results with those reported in the literature.

Works	Methods	Accuracy	F1-Score	Recall
[22]	VGG16	90.54	92.9	98.7
[23]	VGG16	87.18	90	96
	VGG19	88.46	91	95
	ResNet50	77.56	84	97
	Inception-v3	70.99	78	84
[8]	CNN (51 layers)	90.5	92.7	96.7
[24]	RetinaNet with Mask RCNN	-	77.5	79.3
[25]	EL	93.91	93.43	92.99
[9]	Ensemble CNN	84.12	88.56	99.23
[26]	Attention-Based DenseNet	92.8	94.3	96.2
[27]	CNN-XGboost	87	87	85
Our model	CapsNet+ Baysian Optimization	95	96.8	98.9

## Data Availability

The data used in this research are publicly available and can be accessed at https://www.kaggle.com/datasets/paultimothymooney/chest-xray-pneumonia (accessed on 1 September 2025).
